# The Acceptability and Efficacy of Electronic Data Collection in a Hospital Neurodevelopmental Clinic: Pilot Questionnaire Study

**DOI:** 10.2196/18214

**Published:** 2021-01-19

**Authors:** Shrujna Patel, Kelsie Ann Boulton, Marie Antoinette Redoblado-Hodge, Angela Papanicolaou, Diana Barnett, Beverley Bennett, Suzi Drevensek, Jane Cramsie, Kalaichelvi Ganesalingam, Natalie Ong, Magdalen Rozsa, Rebecca Sutherland, Marcia Williamsz, Izabella Pokorski, Yun Ju Christine Song, Natalie Silove, Adam John Guastella

**Affiliations:** 1 Autism Clinic for Translational Research Brain and Mind Centre, Children’s Hospital Westmead Clinical School Faculty of Medicine and Health, University of Sydney Camperdown Australia; 2 Child Neurodevelopment and Mental Health Team Brain and Mind Centre, Children’s Hospital Westmead Clinical School Faculty of Medicine and Health, University of Sydney Sydney Australia; 3 Child Development Unit The Children’s Hospital at Westmead Sydney Children’s Hospital Network Westmead Australia

**Keywords:** electronic data collection, family-centered care, hospital-based data collection

## Abstract

**Background:**

There is a growing need for cost-efficient and patient-centered approaches to support families in hospital- and community-based neurodevelopmental services. For such purposes, electronic data collection (EDC) may hold advantages over paper-based data collection. Such EDC approaches enable automated data collection for scoring and interpretation, saving time for clinicians and services and promoting more efficient service delivery.

**Objective:**

This pilot study evaluated the efficacy of EDC for the Child Development Unit, a hospital-based diagnostic assessment clinic in the Sydney Children’s Hospital Network. Caregiver response rates and preference for EDC or paper-based methods were evaluated as well as the moderating role of demographic characteristics such as age, level of education, and ethnic background.

**Methods:**

Families were sent either a paper-based questionnaire via post or an electronic mail link for completion before attending their first on-site clinic appointment for assessment. A total of 62 families were provided a paper version of the questionnaire, while 184 families were provided the online version of the same questionnaire.

**Results:**

Completion rates of the questionnaire before the first appointment were significantly higher for EDC (164/184, 89.1%) in comparison to paper-based methods (24/62, 39%; *P*<.001). Within the EDC group, a vast majority of respondents indicated a preference for completing the questionnaire online (151/173, 87.3%), compared to paper completion (22/173, 12.7%; *P*<.001). Of the caregiver demographic characteristics, only the respondent’s level of education was associated with modality preference, such that those with a higher level of education reported a greater preference for EDC (*P*=.04).

**Conclusions:**

These results show that EDC is feasible in hospital-based clinics and has the potential to offer substantial benefits in terms of centralized data collation, time and cost savings, efficiency of service, and resource allocation. The results of this study therefore support the continued use of electronic methods to improve family-centered care in clinical practices.

## Introduction

Electronic data collection (EDC) has been at the center of debate about the future of 21st century health care [1-3]. Such approaches have the potential to save billions in health care costs through improved data capture and clinical service responses that allow for more efficient patient-centered care [4-7]. There has, however, been a slow uptake of EDC approaches in clinical services globally, and limited evidence of successful technology integration in public hospital settings [5,8-10]. For instance, in most hospital-based clinics across Australia, data collection is largely paper based. Electronic medical records are being introduced in hospitals; however, this process has been slow and data entry into electronic medical records remains less than systematic [11]. As a result, a recent inquiry report by the Australian Government Productivity Commission suggested that Australia was falling behind in utilizing health care data for data linkage between health services for research purposes [12].

Despite these issues, EDC offers many benefits, warranting its evaluation in public health service settings. EDC provides the opportunity to engage families in more efficient services. For instance, patients can conveniently access forms and staff require less time to monitor, analyze, and interpret the gathered data, allowing for swift provision of feedback to patients and families [13,14]. In addition to this increased efficiency, EDC has been shown to result in fewer human errors in data processing and enables collection of data from a broader geography, increasing completeness of data collation and freeing clinical service resources for other needs, ultimately improving service outcomes [15,16]. The collection and integration of large amounts of data may then be better used to support clinical and research services that operate across rural and remote settings, where on-site attendance can be difficult [15-17].

One public health setting that could benefit considerably from EDC is child diagnostic and assessment services, specifically those clinics that assess children with neurodevelopmental concerns. These neurodevelopmental clinics aim to provide assessments at the earliest possible time in a child’s development to increase the opportunity for earlier assessment, diagnosis, and intervention [18-20], with growing evidence that early intervention is associated with better long-term outcomes for the child and family [21,22]. Currently, however, public services are inundated with assessment requests, long wait lists, and limited resources to complete these tasks. These clinics typically do not use EDC, relying instead on pencil and paper for the vast majority of assessments. These public neurodevelopmental clinics are also more likely to provide services to a higher proportion of children and caregivers from disadvantaged backgrounds, those of lower socioeconomic status, and a higher proportion of linguistically diverse and indigenous communities in comparison to private clinical practices. It is, therefore, important to evaluate the utility of EDC in services that attend to these diverse patient populations.

Prior research has shown that demographic factors, such as age, socioeconomic status, level of education, language, and ethnicity may influence the completion of online data collection [23]. For example, socioeconomic deprivation (measured by the Scottish Index of Multiple Deprivation) and age (>70 years) have been associated with poorer completion of EDC in an orthopedic clinic in Scotland. Socioeconomic deprivation and age were both independently associated with lack of internet access [24], which may have contributed to the study findings. Similarly, a study of orthopedic surgery patients in California found that patients who were older (>75 years), of Hispanic or Black ethnicity, and had Medicare or Medicaid insurance were less likely to complete EDC patient-reported outcome surveys [25]. The authors argued that internet use is less prevalent among older patients, who formed much of the Medicare group. Additionally, Medicaid insurance includes low-income and vulnerable families who may not have had internet access to complete EDC surveys [25].

This study aimed to evaluate an initial pilot for EDC in one of Australia’s busiest child diagnostic and assessment services, the Child Development Unit (CDU) at The Children’s Hospital Westmead, part of the publicly funded Sydney Children’s Hospital Network, Australia. The CDU assesses approximately 600 children per year, referred by pediatricians, who present with complex neurodevelopmental problems. The CDU provides multidisciplinary neurodevelopmental assessments to the state of New South Wales, with some families attending from regional and rural areas, and a high proportion of families from culturally and linguistically diverse backgrounds. Assessment in the CDU begins with gathering information on family and developmental history, via a questionnaire completed by caregivers before attending their first appointment. The CDU has traditionally mailed a paper version of this questionnaire to families prior to their appointment, with families asked to post the completed questionnaire back to the clinic ahead of their appointment so that clinicians can be prepared for the on-site assessments. Clinicians have noted, however, that response rates have been consistently low, with less than 50% of families returning the questionnaire. Such problems lead to delays in terms of clinicians needing to complete and interpret the questionnaire with the family during their appointment.

To the best of our knowledge, this is the first study to assess EDC in a child development clinic. In this pilot study, we digitized the CDU’s caregiver questionnaire into a format that families could access via email and complete electronically in a secure, convenient, and efficient manner. We aimed to examine whether this would improve response rates for the questionnaire when compared to the paper version. We also investigated whether families preferred the electronic modality over the paper version, and the demographic characteristics that were associated with these preferences.

## Methods

### Setting

A total of 246 families who had an appointment in 2018-2019 with the CDU for the initial developmental assessment of their child were invited to participate in this study. Participants were consecutively recruited into this research study using opt-out informed consent methods. This study was approved by the Sydney Children’s Hospital Network Ethics Committee (LNR/17/SCHN/293). The first 62 families entered into this study were sent the paper questionnaire by post. Subsequently, the service transitioned to EDC methods, and a further 184 families were sent the questionnaire via email. No family declined to participate in this study.

Children are referred to the CDU for assessment of complex neurodevelopmental difficulties, including possible autism spectrum disorder, intellectual disabilities, global developmental delay, or specific learning disorders. Prior to their appointment at the CDU, families are expected to complete a 6-page questionnaire, covering demographic information, family history, and child developmental history. The questionnaire has a Flesch Reading Ease score of 76.7, indicating fairly easy readability that should be understood by 12- to 15-year olds [26]. These data enable the CDU to assemble a suitable team and prepare the relevant assessment measures before the appointment. Originally completed by families on paper, we digitized the questionnaire, creating a digital form on the Research Enterprise Data CAPture (REDCap) platform. REDCap is an electronic data capture tool endorsed by the University of Sydney for the secure collection of all research data [27,28]. REDCap is designed to support data capture for research studies and allows questionnaires to be emailed to families ahead of their appointment. Families can click on the link provided in the email invitation to open the questionnaire. Data are automatically saved in REDCap as they are entered in the online form. Once the family exits the form, it is immediately available for clinicians to view.

### Procedure

**Figure 1:**
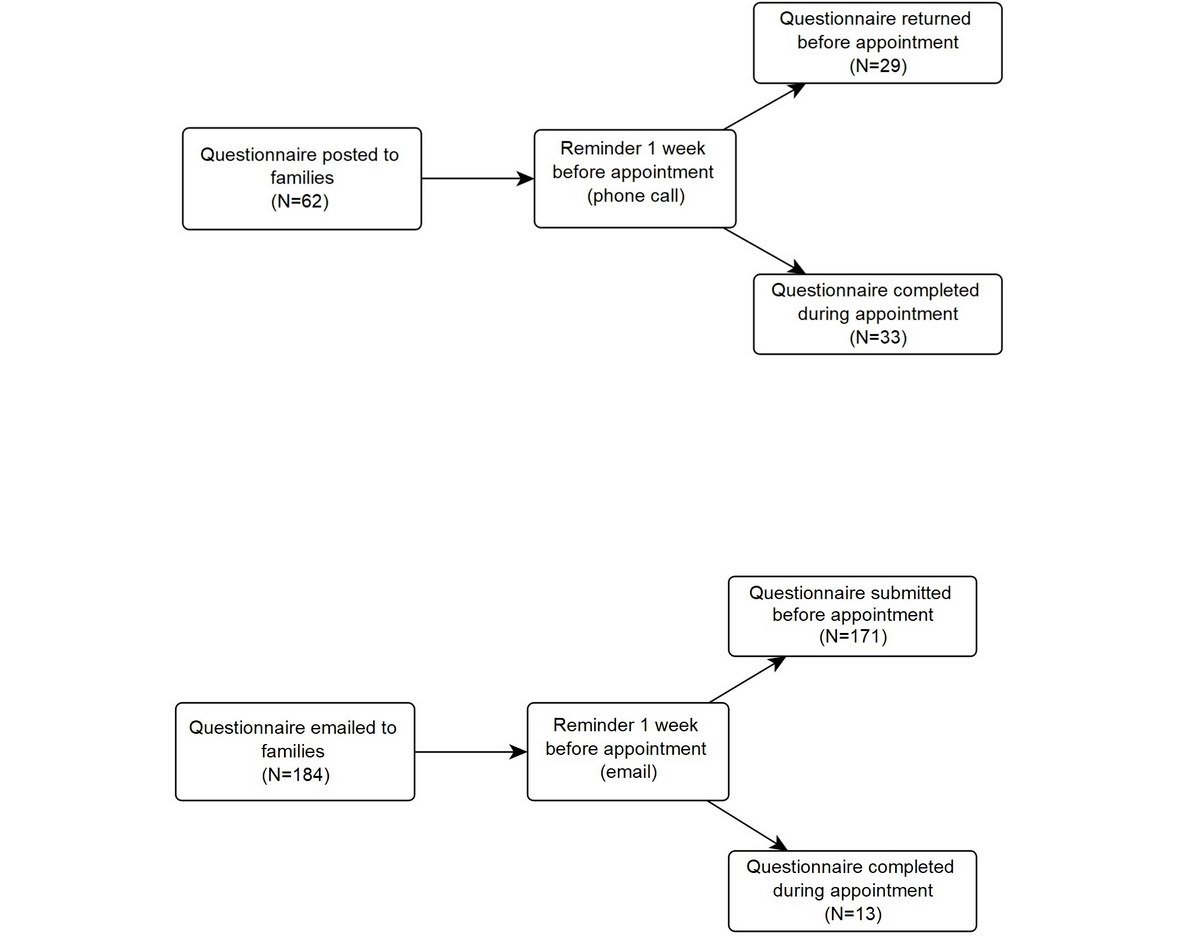
Flow chart of procedures for questionnaire completion modes and response rates for each mode. Numbers in parentheses describe the response rate for each mode.

As per the existing CDU procedure, families were advised of their scheduled appointment via phone. During this phone call, they are informed that a questionnaire will be posted to them and are instructed to complete it and post it back to the clinic. A week before their appointment, families who have not returned the questionnaire via post receive a reminder phone call to do so. Those families who have not returned the questionnaire by the time of their appointment are required to complete it on the day of their appointment with a member of the clinical team (social worker or clinical nurse consultant). In this study, we implemented a pilot REDCap procedure for completion of the questionnaire. Families were advised by phone of their appointment confirmation and told to expect an email inviting them to complete the questionnaire on the REDCap platform. A week before their appointment, an email reminder was sent automatically via REDCap to families who had not completed the questionnaire. Families who had not completed the questionnaire by the time of their appointment were required to complete it on the day of their appointment with a member of the clinical team (social worker or clinical nurse consultant). The existing CDU procedure and pilot REDCap procedure are outlined in Figure 1.

### Statistical Analyses

Response rates were compared for questionnaires sent to families via post (paper completion rates) and those sent to families via email (EDC completion rates). Differences in questionnaire response rates for the postal (existing CDU procedure) and EDC (pilot REDCap procedure) groups were analyzed using chi-square tests.

Within the EDC group, we conducted additional chi-square tests to assess questionnaire modality preference (online or paper). To investigate the influence of demographic characteristics (age, primary language spoken, and highest level of education of caregiver completing questionnaire) on questionnaire modality preference in the EDC group, independent samples *t* tests and chi-square tests were used. Additionally, given that the CDU services families from diverse ethnic backgrounds, chi-square tests were used to assess questionnaire modality preference in respondents who requested an interpreter for the developmental assessment, and respondents who identified as being Aboriginal or Torres Strait Islander (ATSI).

## Results

### Study Population

Data were collected from 246 families across 2018 and 2019. Postal data were collected on 62 families seen in a 3-month period between June and August 2018, and online data were collected on 184 families seen between March and November 2019. Table 1 shows the distribution of responding across the postal and EDC groups.

**Table 1:** Questionnaire response rates for the postal (N=62) and EDC (N=184) groups.

Questionnaire completion status	Postal, n (%)	EDC^a^, n (%)
Completed before appointment	24 (38.7)	164 (89.1)
Partially completed before appointment	5 (8.1)	7 (3.8)
Completed during appointment	33 (53.2)	10 (5.4)
Partially completed during appointment	0 (0.0)	3 (1.6)

^a^EDC: electronic data collection.

### Differences in Response Rates Between the Postal and EDC Groups

As shown in Table 1, there was a significantly higher response rate in the EDC group (164/184, 89.1%) compared to the postal group (24/62, 39%; χ^2^_3_=78.8, *P*<.001). There was no variability in the number of partially completed responses between the EDC (10/184, 5.4%) and postal (5/62, 8%) groups (χ^2^_1_=0.2, *P*=.17).

### Modality Preference

As shown in Table 2, families in the EDC group reported a significantly greater preference for completing and submitting the questionnaire online (151/173, 87.3%) compared to via post (22/173, 12.7%; χ^2^_1_=96.2, *P*<.001). This preference did not vary as a function of when the questionnaire was completed (ie, prior to or during appointment) or the amount of the questionnaire completed (ie, partial or full completion).

**Table 2:** Modality preference for questionnaire completion in the EDC^a^ group.^b^

Questionnaire completion status	Paper preference, n (%)	Online preference, n (%)
Completed before appointment	21 (12.1)	143 (82.7)
Partially completed before appointment	0 (0.0)	1 (0.6)
Completed during appointment	1 (0.6)	7 (4.0)

^a^EDC: electronic data collection.

^b^Preference data missing for 11/184 families (6% of online sample). Percentages reported on the 173 respondents with completed preference data.

### Influence of Demographic Characteristics on Questionnaire Modality Preference

Table 3 displays key demographic characteristics for individuals who completed the questionnaire in the EDC group. Caregiver ages ranged from 24 to 72 years (mean 37.41 [SD 7.05]) and most respondents (145/173, 83.8%) reported English as the main language spoken at home, either alone or in conjunction with a second language. Education level of caregivers who completed the questionnaires was stratified into nontertiary education (high school/vocational/trade) or tertiary education (undergraduate degree/postgraduate degree). In considering the influence of these characteristics on modality preference, the age of the person completing the questionnaire did not influence preference for online compared to paper completion, *t*_167_=0.99, *P*=.32, nor did the primary language spoken by the person completing the questionnaire, χ^2^_2_=2.9, *P*=.24. However, our results revealed an association between education level and questionnaire modality preference, χ^2^_2_=4.2, *P*=.04. Overall, those individuals who had received tertiary education were less likely to report a preference for completing the questionnaire on paper, relative to those individuals who had received nontertiary education. Looking at the education levels of individuals who reported a preference for completing the online questionnaires, the opposite pattern was observed, such that a higher proportion of tertiary educated individuals reported a preference for online completion, compared to nontertiary educated individuals.

**Table 3:** Demographic characteristics by questionnaire modality preference in the EDC^a^ group.

Characteristic		Paper preference	Online preference	*P* value^b^
Age (years), mean (SD)		36.02 (7.02)	37.62 (7.06)	.32
**Primary language spoken, n (%)^c^**				.24
	English only	11 (6.4)	86 (49.7)	
	English and other language	5 (2.9)	43 (24.9)	
	Other language only	6 (3.5)	20 (11.6)	
**Highest level of education, n (%)^d^**				.04
	Nontertiary	15 (8.7)	66 (38.2)	
	Tertiary	7 (4.0)	81 (46.8)	

^a^EDC: electronic data collection.

^b^*P* value for independent samples *t* test (age) and chi-square test of independence (primary language spoken and highest level of education) for any group differences.

^c^Two respondents (2/173, 1.2%) did not provide information about primary language spoken at home.

^d^Four respondents (4/173, 2.3%) did not provide information about highest level of education.

Table 4 shows questionnaire modality preferences (online or paper) for caregivers who requested an interpreter for the assessment (10/173, 5.8%), and caregivers of ATSI origin (10/173, 5.8%). Within both subgroups of caregivers, there was no statistically significant difference in the number of families preferring online or paper completion (*P*=.53 and .21, respectively).

**Table 4:** Questionnaire modality preference for caregivers requesting an interpreter and ATSI caregivers in the EDC^a^ group.^b^

Characteristic	Paper preference, n (%)	Online preference, n (%)	*P* value
Families requesting interpreter	4 (2.3)	6 (3.5)	.53
ATSI^c^ origin	3 (1.7)	7 (4.0)	.21

^a^EDC: electronic data collection.

^b^Ten respondents (10/173, 5.8%) requested an interpreter for the assessment. A further 10 respondents (10/173, 5.8%) identified as being ATSI. There was no overlap between these subgroups of respondents.

^c^ATSI: Aboriginal or Torres Strait Islander.

## Discussion

The results of this study show that EDC was associated with significantly increased questionnaire completion rates (*P*<.001) from caregivers prior to attending their first appointment. Response rates from EDC were more than double the rate from paper-based data collection methods. This overall superior completion rate was shown across families of different ethnic backgrounds and from caregivers with different education levels. Almost 90% (151/173, 87.3%) of caregivers who completed EDC reported a continued preference for using EDC over paper-based methods. This preference did not vary as a function of age, primary language spoken, or belonging to a minority subgroup. Consistent with previous findings, however, a higher level of education (tertiary compared to nontertiary) appeared to be associated with modality preference [23]. Of those caregivers who indicated a preference for the paper version, a greater proportion reported nontertiary education as their highest level. Overall, this pilot study supports the continued evaluation of EDC to improve efficiency, cost, and clinical and research services in public hospital–based child development clinics and supports its utility across diverse education levels and cultural groups [29].

Our finding of reduced questionnaire completion rates when paper-based data collection methods were used align with the clinical experiences of the CDU team, with staff reporting a long history of low response rates for the paper version of the questionnaire. This low response rate results in added clinical burden, as clinicians are required to complete the questionnaire with families at the time of their on-site appointment. This is far from ideal, given the logistics involved in preparing for each on-site assessment. For instance, the CDU carries out approximately 15 comprehensive assessments per week, spanning 1-3 full days. Assessments include tests of intelligence, developmental delay, language, neuropsychological assessments, comprehensive parent interviews, and medical examinations. Assessments are complex, requiring specific rooms, materials, and team members to be organized in advance. Without receiving the completed questionnaire prior to a family’s appointment, the team of multidisciplinary clinicians are unable to adequately prepare for the type of assessment required in advance of the appointment. Our findings indicate that response rates are markedly improved when EDC is used, thereby giving the clinical team time to adequately prepare for assessments and optimizing time with families during assessments.

The increased response rates for the online questionnaire may be related to the increased preference seen for the electronic mode of completion [30-32]. Of note, in this study, we found that respondent age did not influence questionnaire preference for EDC. This finding is in contrast to previous studies that have reported a link between respondent age, response rates, and modality preferences [24,25,33]. Past studies have reported, however, that older age (eg, >60 years) is associated with greater preference for paper-based methods [33]. Given our sample principally comprised parents of young children, with a mean age of 37 and only 1 respondent above the age of 60, future studies may need to evaluate the utility of EDC in this service where primary caregivers are older (eg, grandparents). Our study also showed that the primary language spoken by the respondent did not influence questionnaire modality preference. Families who spoke a primary language other than English did not show differential preferences. Moreover, for 2 minority subgroups, namely, families who requested an interpreter for the assessment and families of ATSI origin, we did not observe an increased preference for paper-based methods compared to EDC. While these findings require replication in larger samples, they indicate that EDC may be suitable for the diverse populations typically serviced by developmental clinics such as the CDU. A more detailed investigation of language and ethnicity, and how these characteristics relate to socioeconomic status, may reveal differences and warrants further investigation [25].

In the small group of respondents who indicated a preference for the paper form, a majority (15/22, 68%) reported nontertiary education as their highest level. It has been shown that mothers with a high-school certificate level education or lower were less likely to use the internet for health-related purposes than those with a tertiary education. This may also be associated with lower socioeconomic status, lower household income, and lack of access to a computer or internet at home [34]. Education level is a known social determinant of health behavior and one that is difficult to address [35,36]. Publicly funded educational programs for vulnerable families may be a useful strategy to assist these families in better understanding their clinical care and options. Moreover, from a practical perspective, an understanding of the families likely to prefer paper forms will allow services such as the CDU to refocus their resources, by providing greater support at service entry to those families who cannot access EDC methods or require assistance from a team member. However, while we observed an association between education level and preference for a paper form, it should be noted that only a small minority of respondents (22/173, 12.7%) indicated preference for a paper version, highlighting the overall acceptability of EDC in this group.

There are some limitations in this study, namely, the relatively small sample size and uneven numbers in the postal and EDC groups. As the study aimed to explore tolerability of EDC, modality preference was only asked of online users. Additionally, the digitized questionnaire was a relatively short measure, taking approximately 15 minutes to complete. Results may differ for larger batteries of questionnaires and this would warrant further investigation in larger sample sizes. While we did not include an economic analysis in this study for EDC methods over paper-based approaches, this is clearly an avenue for future research. Such research would highlight the potential economic value of investing in high-quality internet-based health services for public settings. Moreover, future research would benefit from examining the feasibility and efficacy of EDC for clinician-collected data and evaluating staff satisfaction with these EDC methods. Such work would demonstrate the feasibility of extending EDC methods beyond patient-collected data in clinical health services such as the CDU.

Overall, this pilot study suggests that EDC is feasible and well accepted in a busy hospital-based clinic and has potential benefits for patient care, clinical practice, and clinical research. The increased response rates for online completion and the increased preference for EDC as opposed to paper forms suggest that EDC platforms may better suit the needs of families accessing these services.

## Abbreviations

ATSI: Aboriginal or Torres Strait Islander
CDU: Child Development Unit
EDC: electronic data collection
REDCap: Research Enterprise Data CAPture

## References

[1] Klecun E (2016). Transforming healthcare: policy discourses of IT and patient-centred care. European Journal of Information Systems.

[2] Lavallee DC, Chenok KE, Love RM, Petersen C, Holve E, Segal CD, Franklin PD (2016). Incorporating patient-reported outcomes into health care to engage patients and enhance care. Health Affairs.

[3] Ovretveit J, Keller C, Hvitfeldt FH, Essén A, Lindblad S, Brommels M (2013). Continuous innovation: developing and using a clinical database with new technology for patient-centred care--the case of the Swedish quality register for arthritis. Int J Qual Health Care.

[4] Wang SJ, Middleton B, Prosser LA, Bardon CG, Spurr CD, Carchidi PJ, Kittler AF, Goldszer RC, Fairchild DG, Sussman AJ, Kuperman GJ, Bates DW (2003). A cost-benefit analysis of electronic medical records in primary care. Am J Med.

[5] Gardner W, Morton S, Tinoco A, Scholle SH, Canan BD, Kelleher KJ (2016). Is It Feasible to Use Electronic Health Records for Quality Measurement of Adolescent Care?. J Healthc Qual.

[6] Staziaki PV, Kim P, Vadvala HV, Ghoshhajra BB (2016). Medical Registry Data Collection Efficiency: A Crossover Study Comparing Web-Based Electronic Data Capture and a Standard Spreadsheet. J Med Internet Res.

[7] Highfill T (2019). Do hospitals with electronic health records have lower costs? A systematic review and meta-analysis. International Journal of Healthcare Management.

[8] Palacio C, Harrison JP, Garets D (2010). Benchmarking electronic medical records initiatives in the US: a conceptual model. J Med Syst.

[9] Park Y, Han D (2017). Current Status of Electronic Medical Record Systems in Hospitals and Clinics in Korea. Healthc Inform Res.

[10] Ovretveit John, Scott T, Rundall TG, Shortell SM, Brommels M (2007). Implementation of electronic medical records in hospitals: two case studies. Health Policy.

[11] Canaway R, Boyle DI, Manski-Nankervis JE, Bell J, Hocking JS, Clarke K, Clark M, Gunn JM, Emery JD (2019). Gathering data for decisions: best practice use of primary care electronic records for research. Med J Aust.

[12] Productivity Commission (2017). Data Availability and Use, Inquiry Report.

[13] Dickinson FM, McCauley M, Madaj B, van den Broek N (2019). Using electronic tablets for data collection for healthcare service and maternal health assessments in low resource settings: lessons learnt. BMC Health Serv Res.

[14] Bodagh N, Archbold RA, Weerackody R, Hawking MKD, Barnes MR, Lee AM, Janjuha S, Gutteridge C, Robson J, Timmis A (2018). Feasibility of real-time capture of routine clinical data in the electronic health record: a hospital-based, observational service-evaluation study. BMJ Open.

[15] Ebert JF, Huibers L, Christensen B, Christensen MB (2018). Paper- or Web-Based Questionnaire Invitations as a Method for Data Collection: Cross-Sectional Comparative Study of Differences in Response Rate, Completeness of Data, and Financial Cost. J Med Internet Res.

[16] Thriemer K, Ley B, Ame SM, Puri MK, Hashim R, Chang NY, Salim LA, Ochiai RL, Wierzba TF, Clemens JD, von SL, Deen JL, Ali SM, Ali M (2012). Replacing paper data collection forms with electronic data entry in the field: findings from a study of community-acquired bloodstream infections in Pemba, Zanzibar. BMC Res Notes.

[17] Bjertnaes O, Iversen HH, Skrivarhaug T (2018). A randomized comparison of three data collection models for the measurement of parent experiences with diabetes outpatient care. BMC Med Res Methodol.

[18] Barbaro J, Dissanayake C (2009). Autism spectrum disorders in infancy and toddlerhood: a review of the evidence on early signs, early identification tools, and early diagnosis. J Dev Behav Pediatr.

[19] Rutter M (2006). Autism: its recognition, early diagnosis, and service implications. J Dev Behav Pediatr.

[20] Koegel LK, Koegel RL, Ashbaugh K, Bradshaw J (2014). The importance of early identification and intervention for children with or at risk for autism spectrum disorders. Int J Speech Lang Pathol.

[21] McConachie H, Diggle T (2007). Parent implemented early intervention for young children with autism spectrum disorder: a systematic review. J Eval Clin Pract.

[22] Ben Itzchak E, Zachor DA (2011). Who benefits from early intervention in autism spectrum disorders?. Research in Autism Spectrum Disorders.

[23] Fan W, Yan Z (2010). Factors affecting response rates of the web survey: A systematic review. Computers in Human Behavior.

[24] Jenkins P, Sng S, Brooksbank K, Brooksbank A (2016). Socioeconomic deprivation and age are barriers to the online collection of patient reported outcome measures in orthopaedic patients. Ann R Coll Surg Engl.

[25] Schamber EM, Takemoto SK, Chenok KE, Bozic KJ (2013). Barriers to completion of Patient Reported Outcome Measures. J Arthroplasty.

[26] Flesch, R (1948). A new readability yardstick. J Appl Psychol.

[27] Harris PA, Taylor R, Thielke R, Payne J, Gonzalez N, Conde JG (2009). Research electronic data capture (REDCap)--a metadata-driven methodology and workflow process for providing translational research informatics support. J Biomed Inform.

[28] Harris PA, Taylor R, Minor BL, Elliott V, Fernandez M, O'Neal L, McLeod L, Delacqua G, Delacqua F, Kirby J, Duda SN, REDCap Consortium (2019). The REDCap consortium: Building an international community of software platform partners. J Biomed Inform.

[29] Duffy JR, Kooken WC, Wolverton CL, Weaver MT (2012). Evaluating patient-centered care: feasibility of electronic data collection in hospitalized older adults. J Nurs Care Qual.

[30] Mlikotic R, Parker B, Rajapakshe R (2016). Assessing the Effects of Participant Preference and Demographics in the Usage of Web-based Survey Questionnaires by Women Attending Screening Mammography in British Columbia. J Med Internet Res.

[31] Olson K, Smyth JD, Wood HM (2012). Does Giving People Their Preferred Survey Mode Actually Increase Survey Participation Rates? An Experimental Examination. Public Opinion Quarterly.

[32] Smyth JD, Olson K, Millar MM (2014). Identifying predictors of survey mode preference. Soc Sci Res.

[33] Fitzgerald D, Hockey R, Jones M, Mishra G, Waller M, Dobson A (2019). Use of Online or Paper Surveys by Australian Women: Longitudinal Study of Users, Devices, and Cohort Retention. J Med Internet Res.

[34] Wen LM, Rissel C, Baur LA, Lee E, Simpson JM (2011). Who is NOT likely to access the Internet for health information? Findings from first-time mothers in southwest Sydney, Australia. Int J Med Inform.

[35] Li J, Mattes E, Stanley F, McMurray A, Hertzman C (2014). Social determinants of child health and well-being. Health Sociology Review.

[36] Li J, Powdthavee N (2015). Does more education lead to better health habits? Evidence from the school reforms in Australia. Soc Sci Med.

